# Numerical Simulation of Stainless Steel-Carbon Steel Laminated Plate Considering Interface in Pulsed Laser Bending

**DOI:** 10.3390/ma12091410

**Published:** 2019-04-30

**Authors:** Zihui Li, Xuyue Wang

**Affiliations:** School of Mechanical Engineering, Dalian University of Technology, Dalian 116023, China; wbzzd@dlut.edu.cn

**Keywords:** laser bending, stainless steel-carbon steel laminated plate, interface, depth of plastic zone, numerical simulation, bending angle

## Abstract

According to ANSYS software and an electron probe experiment, a multi-layer finite element model (FEM) of pulsed laser bending of stainless steel-carbon steel laminated plate (SCLP) including interfaces has been established. Compared with a single-layer stainless steel plate (SLSP), based on a temperature gradient mechanism considering the depth of the plastic zone, the influence of the interfaces and carbon steel layer in the model of the SCLP on the bending angle has been studied by analyzing the distributions of the temperature field, stress field and strain field in the thickness direction. The simulation results show that the temperature of the SCLP in the thickness direction is lower than that of the SLSP due to interfacial thermal resistance of the interface and fast heat conduction of the carbon steel layer, resulting in a smaller depth of the plastic zone of the SCLP defined by the recrystallization temperature. Affected by the temperature distribution, the plastic stress and strain of the SCLP in the plastic zone are smaller than those of the SLSP, leading to a smaller bending angle of the SCLP. When the laser power is 140 W, the scanning speed is 400 mm/min, the defocus distance is 10 mm, and the scanning time is 1, the bending angle of the SCLP is 1.336°, which is smaller than the bending angle 1.760° of the SLSP. The experimental verifications show that the maximum error of the bending angle is 3.74%, which verifies that the model of laser bending is usable and contributes to refining the laser bending mechanism of the SCLP.

## 1. Introduction

SCLP is manufactured by combining the cladding layer (stainless steel layer) and the matrix (carbon steel layer) with an explosion, rolling and other processes. As a new kind of laminated metal composite plate, due to the corrosion resistance and high-temperature resistance of 304 stainless steel, and the high heat conductivity and high strength of Q235 carbon steel [1], SCLP has replaced SLSP for useful prospects in the corrugated bulkhead of aircraft and shipbuilding industries. Compared with homogeneous materials, SCLP has different properties in each layer, resulting in an uneven distribution of temperature and stress inside the SCLP. Therefore, it is more difficult to control the bending angle of the SCLP.

The corrugated bulkhead has lots of bending structures. In many bending processes, laser bending has no use for molds and external forces [2], which is suitable to form plates with high precision and good quality by adjusting process parameters [3]. Due to the complexity of the material parameters and the mutual effect of laser and material, many scholars pay attention to deformation analysis during laser bending. In terms of simulation, based on ANSYS software, Liu et al. [4] developed a model including fifteen particles of SiC_p_ to investigate the influence of particle distributions on deformation behavior of an aluminum matrix composite during laser forming. Shen et al. [5], using the finite element method, investigated the mechanical deformation of bilayer plates including only a ceramic layer and a metal layer in laser forming. Shen found that top and bottom surfaces have residual tensile stress and the interface between two layers has residual compressive stress. Kant et al. [6] carried out a numerical study on the deformation mechanism and bending behavior of a magnesium alloy plate with a large bending angle during multi-scan laser bending. Kant found that thermal stress decreased with an increase in scanning time due to the increase of temperature and the decrease of restraint. Song [7] only analyzed the temperature distributions of a laminated metal plate and a single layer metal plate using a plasma arc and ANSYS software. Song concluded that the temperature gradient of a laminated metal plate in the thickness direction is smaller than that of single metal plate, while the heat-affected zone of the laminated plate in the perpendicular direction of scanning path is wider than that of single layer plate with same process parameters. In terms of the experiment, Carey et al. [8] investigated the effect of a number of variables including stacking sequence and fiber layer parameters on laser forming for fiber metal laminates. Maji et al. [9] used a response surface methodology to determine the relationship between the bending angle of an AISI 304 stainless steel plate and the process variables and analyzed statistically the effects of process parameters. Ma et al. [10] measured the grain size of the carbon steel layer in the bending zone of SCLP by image processing software from the perspective of material detection. Ma found that the increase in grain size caused thermal expansion thickening and extrusion thickening in the laser bending process, which is the main reason for the thickening phenomenon. Gisario et al. [11] achieved sharp bending angles of Titanium Grade-2 plates with small fillet radii by adjusting auxiliary bending device and processing parameters. However, the research on laser bending was mainly focused on a single-component metal plate, brittle material and metal matrix composite. There are few numerical studies on metal laminates composed of different materials. Furthermore, many studies have simplified the interface of the laminated plate to an ideal interface without thickness, while the actual bonding interface is a transition layer with a certain thickness. The modeling of the transition layer is key to accurately simulate laser bending SCLP. Besides, considering the transition layer and carbon steel layer, it is significant to establish the deformation mechanism that is suitable for the SCLP to explain the deformation process from the temperature field to the displacement field. Therefore, it is particularly important to accurately build the model and supplement the laser bending mechanism of the SCLP considering the interface.

In this work, based on the ANSYS software and electron probe experiment, a multi-layer FEM of the SCLP including the interfaces with the thickness of 7 μm has been established to simulate the pulsed laser bending process. Then, according to the temperature distribution along the thickness direction of the plate, the depth of the plastic zone is delimited by the recrystallization temperature. Considering the influence of the interfaces and carbon steel layer, the heat conduction process from stainless steel to carbon steel is analyzed in detail. By an analysis of the temperature field, the stress and strain fields of the SCLP are investigated along the thickness direction compared with the SLSP, focusing on the effect of interfaces and the carbon steel layer on the bending angle of the SCLP. Based on the analysis of stress and strain fields, the deformation behavior of the SCLP bending during laser forming is explained, which is consistent with the displacement field. The bending angle of the SCLP is predicted with great accuracy, which provides the bases of theory and experiment for the laser bending of SCLP.

## 2. Methods

### 2.1. Finite Element Equations of Heat Conduction

Along the scanning path, the laser heat source moves at a constant speed to heat the surface of the plate. Assuming that the laser heat source obeys a Gaussian distribution, the power density formula of the pulsed laser can be written as:

(1) $$I(x,y)=AI_{0}f(x,y)g(t)$$

where A is the absorptivity of the SCLP (about 0.25), $I_{0}$ is the power density at the laser spot center, f(x,y) is the spatial distribution of pulsed laser, and g(t) is the time distribution of pulsed laser. For the fundamental mode of Gauss beam, f(x,y) and g(t) can be expressed as:

(2) $$f(x,y)=\exp\left\{-2\left[{\left(x-x_{0}\right)}^{2}+{\left(y-y_{0}\right)}^{2}\right]/r_{0}^{2}\right\}$$

(3) $$g(t)=\left\{ \begin{array}{l} 1\;\;\;\;\;\;0\leq t\leq t_{p}\\ 0\;\;t_{p}<t\leq 1/f \end{array}\right.$$

where $x_{0}$ and $y_{0}$ are the laser spot center’s coordinates, x and y are the current coordinates in laser spot area, $r_{0}$ is the laser spot’s radius, t is the run time, $t_{p}$ is the pulse width (2 ms), and f is the frequency of the pulsed laser (40 Hz).

The material parameters of each layer are assumed to be continuous and isotropic, and the parameters change with temperature. Therefore, the transient temperature field *T(x,y,z,t)* satisfies the Fourier law and conservation of energy under the rectangular coordinate. When the laser beam heats the laminated plate, the heat conduction equation is as follows:

(4) $$k_{j}\left(\frac{\partial^{2}T_{j}}{\partial x^{2}}+\frac{\partial^{2}T_{j}}{\partial y^{2}}+\frac{\partial^{2}T_{j}}{\partial z^{2}}\right)=\rho_{j}c_{j}\overset{\cdot }{T_{j}}$$

where $T_{j}$ is the transient temperature of the j-th layer, $\rho_{j}$, $c_{j}$ and $k_{j}$ are the density, specific heat capacity and heat conductivity of j-th layer separately, and *j* = 1, 2, 3, …n. Assuming that the temperature and thermal flux of each layer at the interface are continuous, the equations are as follows:

(5) $$T_{j}(x,y,z,t)=T_{j+1}(x,y,z,t)$$

(6) $${-k_{j}\frac{\partial T_{j}}{\partial z}|}_{z=h_{j}}={-k_{j+1}\frac{\partial T_{j+1}}{\partial z}|}_{z=h_{j}}.$$

There is heat convection and heat radiation between the SCLP and surrounding environment, so the boundary condition can be expressed as follows:

(7) $$-k\cdot \partial T/\partial n=h_{e}(T-T_{0})$$

where k is heat conductivity, and $h_{e}$ is the equivalent heat transfer coefficient (30 W·m^−2^·K^−1^).

(8) $$h_{e}=h_{c}+h_{r}$$

(9) $$h_{r}=\varepsilon \sigma (T_{w}+T_{0})(T_{w}^{2}+T_{0}^{2})$$

where $h_{c}$ is convective heat transfer coefficient, $h_{r}$ is radiative heat transfer coefficient, ε is heat radiation rate, σ is Stefan-Boltzmann constant, $T_{w}$ is the temperature of the work piece at the boundary, and T is the temperature of the work piece.

Initial condition:

(10) $${T|}_{t=0}=T_{0}$$

where $T_{0}$ is room temperature (293 K).

As the temperature, rate of heat flow, internal energy and thermal boundary conditions of the SCLP change obviously with time in the process of laser bending, Equation (4) is calculated by the Galerkin method. For nonlinear thermal analysis, the equation of heat balance of the SCLP is obtained as follows:

(11) $$\left[C(T)\right]\left\{\overset{\cdot }{T}(t)\right\}+\left[K(T)\right]\left\{T(t)\right\}=\left\{Q(t)\right\}$$

where [C(T)] is the special heat capacity matrix, {T(t)} is the vector of node temperature, $\left\{\overset{\cdot }{T}(t)\right\}$ is the derivative of the node’s temperature with respect to time, [K(T)] is the heat conduction matrix, and {Q(t)} is the vector of node’s heat flow rate.

The backward difference method is used for the discretization of Equation (11), and the governing equation of the transient temperature field is obtained as follows:

(12) $$\left(\left[K(T)\right]+\frac{\left[C(T)\right]}{\Delta t}\right){\left\{T\right\}}_{t}={\left\{Q\right\}}_{t}+\frac{\left[C(T)\right]}{\Delta t}{\left\{T\right\}}_{t-\Delta t}$$

### 2.2. FEM of SCLP Considering Interface

**Figure 1:**
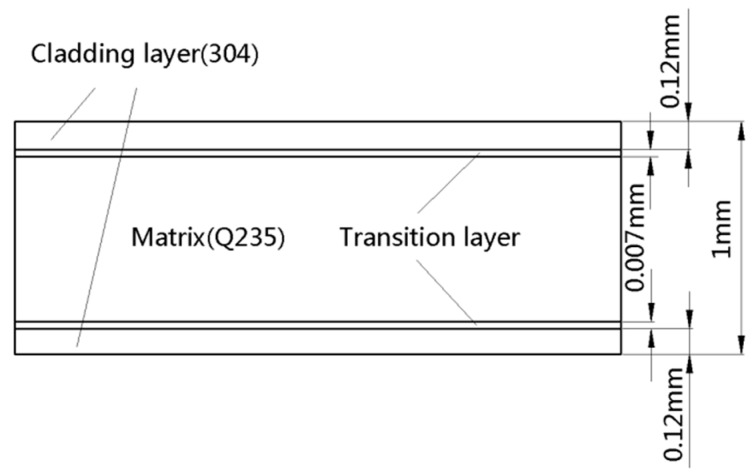
Schematic of the SCLP.

**Figure 2:**
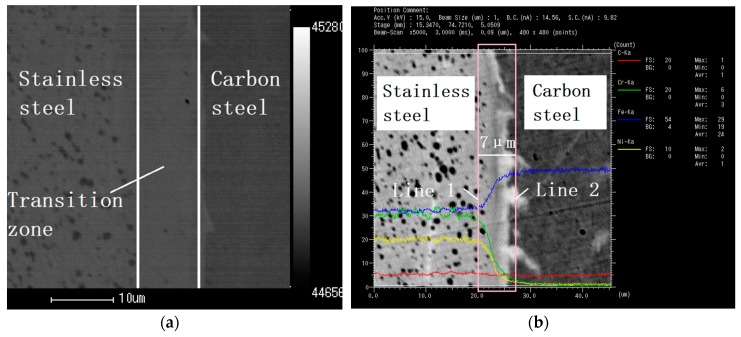
Metallographic microscope observation of the transition layer: (**a**) Metallographic micrograph (5000×); (**b**) electron probe microanalysis (5000×).

**Figure 3:**
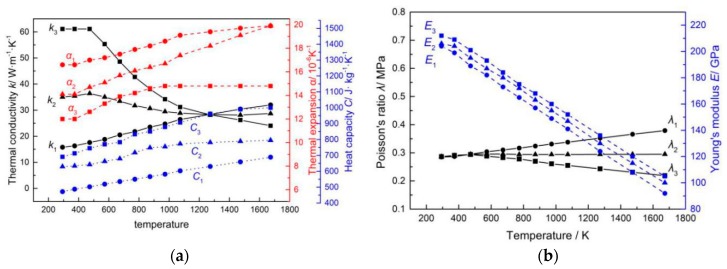
Material parameters of stainless steel and carbon steel: (**a**) Thermodynamic properties; (**b**) mechanical properties.

Under the coordinates of Cartesian, the geometrical model of the SCLP with a size of 60 mm × 50 mm × 1 mm is built according to the real workpiece as shown in Figure 1. The model includes five layers: the upper and lower stainless steel layers, the upper and lower transition layers and the carbon steel layer. Among them, the interface is regarded as a transition layer with a certain thickness. Based on the process criterion, the thicknesses of stainless steel layer, transition layer and carbon steel layer are 120 μm, 7 μm and 746 μm, separately. Specifically, the thickness of the transition layer is measured using EPMA-1600 electron probe. In Figure 2a, there is a dark strip area with a width of approximate 7 μm at the interface of the SCLP, which is the transition layer between the stainless steel layer and the carbon steel layer. Based on the element count intensity of Figure 2b, line 1 and line 2 are the positions where the element distribution changes significantly and becomes gentle, respectively. The distance between the two lines is the thickness of the transition layer, which is 7 μm. Considering the temperature parameters, the thermal and mechanical properties of stainless steel layer, the transition layer and the carbon steel layer are given in Figure 3. Material properties of stainless steel and carbon steel are obtained in the handbook [12]. Material properties of the transition layer are calculated through the equivalent method. Based on the bonding mechanism of the transition layer, the equivalent method considers that the transition layer is a mixture of stainless steel and carbon steel. Therefore, according to the effective medium theory, the Turner formula, the Mori-Tanaka method and mixture law, the equivalent properties of the transition layer are calculated by the following theoretical formulas [13]:

(13) $$k_{c}=\frac{1}{4}\left[k_{1}\left(3V_{1}-1\right)+k_{2}\left(3V_{2}-1\right)+\sqrt{{\left[k_{1}\left(3V_{1}-1\right)+k_{2}\left(3V_{2}-1\right)\right]}^{2}+8k_{1}k_{2}}\right]$$

(14) $$\alpha_{c}=\left[\alpha_{1}E_{1}V_{1}(1-2\lambda_{2})+\alpha_{2}E_{2}V_{2}(1-2\lambda_{1})\right]/\left[E_{1}V_{1}(1-2\lambda_{2})+E_{2}V_{2}(1-2\lambda_{1})\right]$$

(15) $$E_{c}=3K_{2}(1-2\lambda_{c})+3(1-2\lambda_{c})K_{2}V_{1}(K_{1}-K_{2})/\left[K_{2}+(1-V_{1})(K_{1}-K_{2})\left(1+\lambda_{2}\right)/3\left(1-\lambda_{2}\right)\right]$$

(16) $$\rho_{c}=\rho_{1}V_{1}+\rho_{2}V_{2}$$

(17) $$\lambda_{c}=\lambda_{1}V_{1}+\lambda_{2}V_{2}$$

(18) $$C_{c}=\left(\rho_{1}V_{1}C_{1}+\rho_{2}V_{2}C_{2}\right)/\left(\rho_{1}V_{1}+\rho_{2}V_{2}\right)$$

where *k_c_*, *k*_1_ and *k*_2_ are the thermal conductivity of transition layer, stainless steel and carbon steel, respectively; *α_c_*, *α*_1_ and *α*_2_ are the thermal expansion coefficient of transition layer, stainless steel and carbon steel, respectively; *K_c_*, *K*_1_ and *K*_2_ are the bulk modulus of transition layer, stainless steel and carbon steel, respectively; *E_c_*, *E*_1_ and *E*_2_ are the elasticity modulus of transition layer, stainless steel and carbon steel, respectively; *λ_c_*, *λ*_1_ and *λ*_2_ are the Poisson’s ratio of transition layer, stainless steel and carbon steel, respectively; and *V*_1_ (46.63%) and *V*_2_ (53.37%) are the volume fraction of stainless steel and carbon steel in the transition layer, respectively.

**Figure 4:**
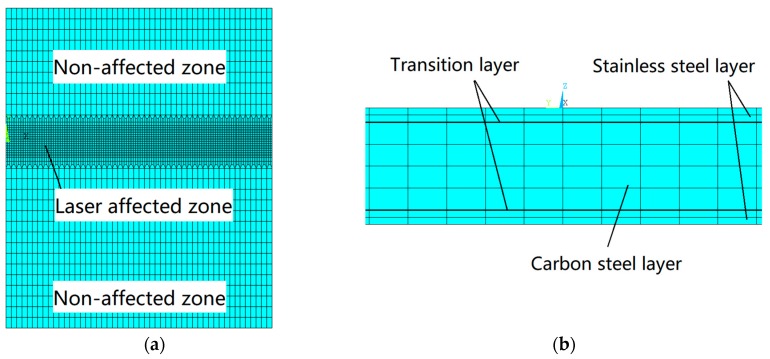
FEM grid partition profile of the SCLP: (**a**) Grid partition in the XOY plane; (**b**) Grid partition in the *Z*-axis direction.

In the numerical simulation of laser bending of SCLP, appropriate mesh density has a significant influence on the precision and efficiency of the simulation. Figure 4 shows the FEM grid partition profile of the SCLP in the XOY plane and in the direction of the *Z*-axis. Especially, as shown in Figure 4a, in the laser affected zone with a width of 6 mm, because of the high temperature and the large stress gradient, the grid of the XOY plane is divided into a smaller size of 1/3 × 1/3 mm. On the contrary, in the non-affected zone, the temperature and stress fields are relatively uniform, so that the grid is chosen as the bigger 1 × 1 mm size. As shown in Figure 4b, in the *Z*-axis direction, the stainless steel layer, the transition layer and the carbon steel layer are divided into two layers, one layer and four layers, respectively. The model of the SCLP includes 61,724 nodes and 60,450 elements. SOLID 70 and SOLID 45 are selected for the analyses of thermology and mechanics. The convective heat transfer radiation between the SCLP and the surrounding environment acts on SURF 152. By ANSYS parametric design language, numerical simulation of the SCLP is achieved during pulsed laser bending.

### 2.3. Temperature Gradient Mechanism Based on Definition of Depth of Plastic Zone

**Figure 5:**
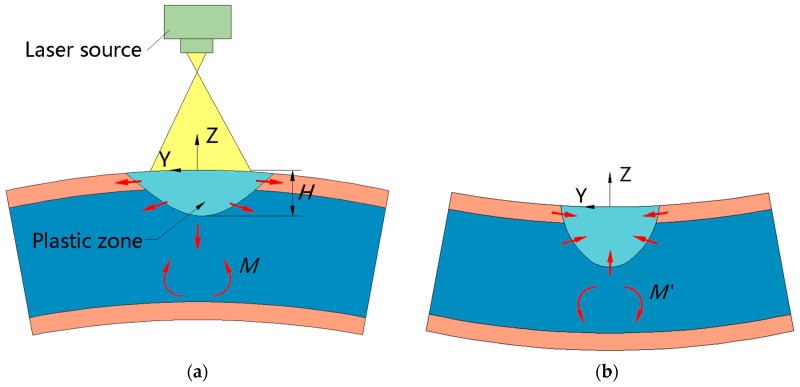
Schematic diagram of temperature gradient mechanism: (**a**) During heating; (**b**) during cooling.

When the laser beam with a fast scanning speed, small diameter and high energy density irradiates on the SCLP’s surface, the upper surface is heated to a high-temperature state, but the temperature of the lower surface is low. Therefore, a large temperature gradient is generated along the thickness direction. In the thickness direction, in the zone where the maximum of temperature exceeds the recrystallization temperature, because of grain growth, the grain boundary migrates and expands outward in the grain structure, leading to a gradual decrease in the area of the grain boundary and the free energy at the interface. The yield limit of the material in this zone is largely reduced so that the material produces large plastic deformation. Hence, the zone where the maximum temperature exceeds the recrystallization temperature is defined as the plastic zone. The depth *H* of the plastic zone is marked in Figure 5a. The recrystallization temperature of the metal is usually calculated as 40% of the melting point of deformed metal in industry.

In Figure 5a, during heating, on account of a steep temperature gradient, the thermal expansion amount of the plastic zone with the depth of *H* is much larger than that of the lower surface, causing negative bending of the SCLP away from laser beam and a small amount of material accumulation on the surface of the SCLP. In the meantime, the moment *M* generated in the lower surface prevents thermal expansion of the plastic zone. However, the SCLP undergoes a positive bending during cooling as shown in Figure 5b. On the one hand, the decrease in the temperature of the upper surface makes the material volume shrink and the yield limit increase so that the material accumulation due to thermal expansion cannot be recovered. On the other hand, although the moment *M*′ of the lower surface of the SCLP prevents positive bending, the temperature of the lower surface increases because of heat conduction, which decreases its yield stress and makes it easy to deform. Consequently, the SCLP produces a forward bending around the scanning line facing the laser beam. From the above temperature gradient mechanism, it can be concluded that, when the depth *H* of the plastic zone is larger, the area and surrounding compressive stress in the plastic zone are larger, leading to accumulation of more material. Next, during cooling, the larger the volumetric contraction of the material in the plastic zone is, the greater the bending angle of the SCLP is.

## 3. Simulations and Analysis

Due to different material properties of each layer, not only temperature, stress and strain distributions of the SCLP are different from those of the SLSP, but also the sharp changes of material properties in the transition layer cause the sharp changes of temperature, stress and strain. Therefore, it is necessary to study the laser bending mechanism of SCLP through ANSYS software. For numerical analysis of laser bending, the process parameters are selected as follows: laser power *P* = 140 W, scanning speed *v* = 400 mm/min, defocus distance *Z* = 10 mm, and scanning time *N* = 1. The simulation time is 100 s, including laser processing time of 7.5 s and cooling time of 92.5 s.

### 3.1. Simulations and Analysis of Temperature Field of SCLP

**Figure 6:**
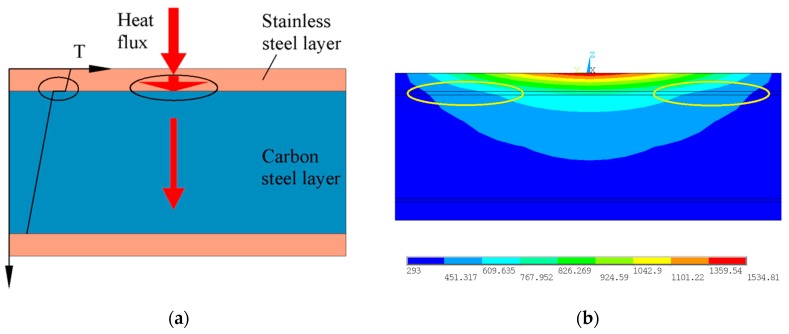
Temperature analysis in the thickness direction: (**a**) Heat transfer diagram at the interface; (**b**) temperature distribution in section.

It is well known that there is interfacial thermal resistance between all different media, so there is thermal resistance at the interface between the stainless steel layer and the carbon steel layer. That is, during the heat conduction process, the temperature at the interface is discontinuous, as shown in Figure 6a. Due to the existence of a 7 μm thick transition layer at the interface, the sharp change phenomenon of temperature is alleviated to a certain extent. The variation value is 7.88 K at the transition layer, as shown in Figure 6b. Compared with SCLP, SLSP has smooth temperature conduction across the interface. As a boundary line between the stainless steel layer and the carbon steel layer, the interface characterizes the difference in heat distribution of the stainless steel layer and the carbon steel layer, and this difference has an effect on the stress and strain fields.

**Figure 7:**
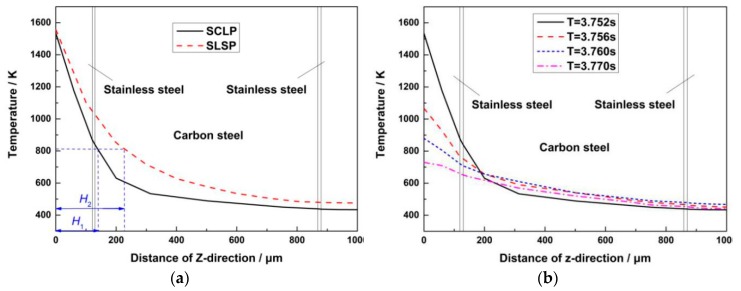
Temperature curve: (**a**) Comparison between the SCLP and the SLSP; (**b**) temperature curves of the SCLP at different moments.

Due to the different thermal properties of stainless steel and carbon steel, the thermal conduction rate of each layer is different during the laser bending process. Figure 7a shows the temperature distribution of the SCLP and the SLSP at the midpoint of the scanning line along the thickness direction under the laser action (T = 3.752 s). The temperature of the SCLP is lower than that of the SLSP in the thickness direction. On the one hand, the temperature of the upper stainless steel layer decreases faster than that of the SLSP, and the temperature from the surface of the SCLP to the transition layer is 1534.81 K to 852.997 K. This is because the heat conductivity of transition layer and carbon steel layer is larger than that of stainless steel layer, resulting in rapid heat diffusion. Therefore, the heat transfer inside the SCLP is faster than that inside the SLSP, and the heat loss is more. On the other hand, the temperature of the carbon steel layer is lower. The reason is that the specific heat capacity of the transition layer and carbon steel layer is larger than that of the stainless steel layer, causing a slow temperature rise. According to the definition of the depth of the plastic zone in Section 2.3, the depths of plastic zone *H*_1_ and *H*_2_ are marked in Figure 7a. It can be found that the depth *H*_1_ of the plastic zone of the SCLP is smaller than *H*_2_ of the SLSP due to the existence of the transition layer and the carbon steel layer. Therefore, the bending angle of the SCLP is smaller than that of the SLSP. Figure 7b shows the temperature curves of the SCLP at different moments in one pulse cycle. It demonstrates that in the interval of a pulsed laser, the existence of the carbon steel layer in the SCLP accelerates the heat conduction in the thickness direction. As a result, due to the heat diffusion into the SCLP, the temperature of the upper stainless steel layer decreases rapidly, and the temperature of the carbon steel layer and lower stainless steel layer increases slowly.

### 3.2. Simulations and Analysis of Stress Field of SCLP

**Figure 8:**
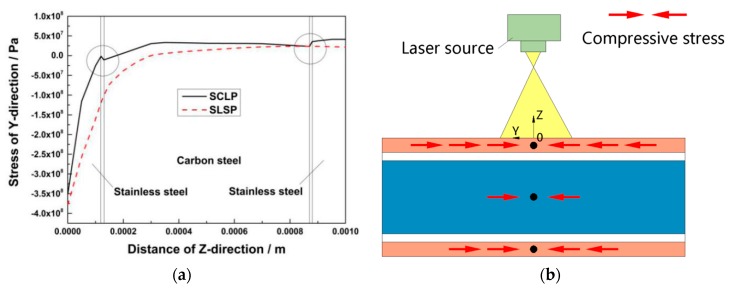
Y-direction stress analysis at 3.752 s: (**a**) Comparison of stress curves of the SCLP and the SLSP; (**b**) schematic diagram of stress distribution of the SCLP.

Figure 8a shows the Y-direction stress curves of the SCLP and the SLSP at the midpoint of the scanning line along the thickness direction under the action of laser (T = 3.752 s). Figure 8a illustrates that there is a sudden increase in the 7 μm thick upper and lower transition layers. The sharp values are 12.19 MPa and 9.33 MPa, respectively. Compared with the SCLP, the SLSP has a continuous and smooth stress curve. For the SCLP, due to the interfacial thermal resistance, the heat stays at the transition layers for a short time, leading to an increase in temperature and thermal expansion. Therefore, the Y-direction compressive stress increases at the interface along the thickness direction. It can be seen from the temperature gradient mechanism that the temperature of the lower transition layer is lower and is less affected by the interfacial thermal resistance. The main reason for the sharp change of the lower transition layer is that the mechanical properties of stainless steel, such as yield strength, are smaller than those of carbon steel, so the force bearing capacity is smaller, which results in that the Y-direction stress of stainless steel is greater than that of carbon steel near the lower transition layer.

In Figure 8a, as the surface of the SCLP expands thermally, the expansion is restricted by the surrounding material, thus causing the area to be effective in compression, and the maximum compressive stress is 352.99 MPa. SLSP has a maximum compressive stress greater than the SCLP due to its higher surface temperature and larger thermal expansion, and the maximum value is 379.54 MPa. In the thickness direction, the depth of the plastic compressive stress of the SCLP is smaller than that of the SLSP. This result corresponds to the temperature field analysis. Figure 8b is the schematic diagram of stress distribution of the SCLP drawn in accordance with Figure 8a, and the number of arrows represents the relative size of compressive stress. Along the thickness direction, the stress of SLSP decreases gradually. However, for the SCLP, because of the mechanical properties of the carbon steel layer, the stress of the carbon steel layer is less than that of the lower stainless steel layer. Consequently, less material in the plastic zone is accumulated. Then, during the cooling stage, the smaller the volumetric contraction of the material is, the smaller the bending angle of the SCLP is. The carbon steel layer accounts for 74.6% of the volume of the SCLP, which plays an important role in bending SCLP.

**Figure 9:**
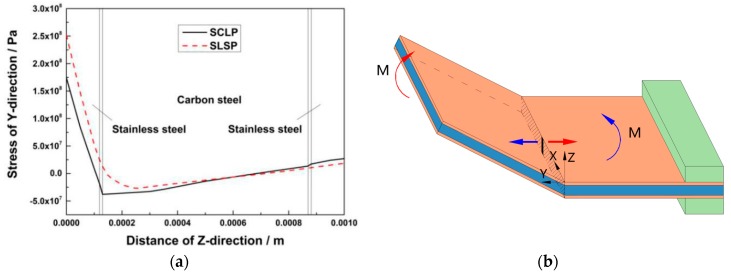
Y-direction stress analysis at 100 s: (**a**) Comparison of stress curves between the SCLP and the SLSP; (**b**) schematic diagram of SCLP bending.

Figure 9a shows Y-direction stress curves of the SCLP and the SLSP at the midpoint of the scanning line along the thickness direction after cooling (T = 100 s). As each action of pulsed laser brings about the stress redistribution of the plate, bending deformation can be understood intuitively from the perspective of residual stress. In Figure 9a, the stainless steel layer in the upper side of the upper transition layer has Y-direction residual tensile stress, while the carbon steel layer in the lower side of the upper transition layer has Y-direction residual compressive stress, resulting in the maximum sharp change of residual stress in the upper transition layer. The maximum sharp change of residual stress is 3.30 MPa. It can also be observed that maximum residual tensile stresses appearing on the upper surfaces of the SCLP and the SLSP are 174.19 MPa and 252.28 MPa, respectively. As the temperature of the SCLP in the plastic zone is lower than that of SLSP, causing a smaller amount of thermal expansion in Section 3.1, the SCLP has a smaller shrinkage and less tensile stress during cooling. Figure 9b is the schematic diagram of SCLP bending drawn in accordance with Figure 9a. It shows that the tensile stress causes bending moment *M* at the free end of the SCLP, which generates the positive bending of the SCLP around the scanning line. Therefore, the magnitude of bending deformation can be reflected by the magnitude of residual tensile stress. According to Figure 9a,b, the bending angle of the SCLP is smaller than that of the SLSP.

### 3.3. Simulations and Analysis of Strain Field of SCLP

**Figure 10:**
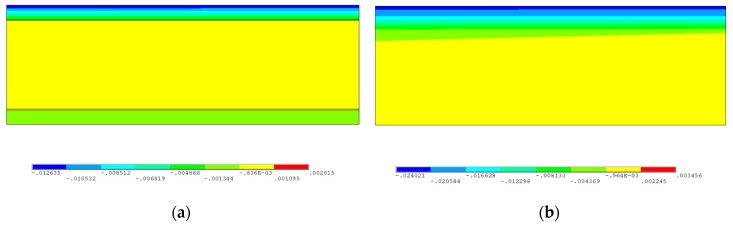
Comparison of Y-direction strain distributions between the SCLP and the SLSP at 100 s: (**a**) SCLP; (**b**) SLSP.

Figure 10a,b show the Y-direction strain distributions of the cross-sections of the SCLP and the SLSP along the scanning line after cooling (T = 100 s), respectively. Due to the thermal expansion under the action of the laser, the compressive stress on the upper surface is larger than that on the lower surface, resulting in a large shrinkage deformation in the Y-direction during cooling, so the compressive strain is also larger. As shown in Figure 10a, the Y-direction strain is mainly concentrated in the upper stainless steel layer. It is indicated that the plastic deformation of the upper stainless steel layer is a main driving force for bending deformation of the SCLP. The maximum residual Y-direction compressive strain of the SCLP appears on the upper surface of the SCLP, and its value is −0.0126. Moreover, corresponding to the sharp changes of temperature and stress at the transition layer, there are sharp changes of strain at the upper and lower interfaces, and the sharp change values are 0.0010 and 0.00027, respectively. Compared with Figure 10a, it can be seen from Figure 10b that the Y-direction strain of the SLSP changes uniformly along the thickness direction, while the compressive strain of the lower stainless steel layer of the SCLP increases due to the change in material properties. Moreover, the compressive strain zone of the SLSP is deeper than that of the SCLP, so that the volume shrinkage of the surface is larger during cooling. Therefore, the bending angle of the SCLP is smaller than that of the SLSP.

### 3.4. Simulations and Analysis of Displacement Field of SCLP

**Figure 11:**
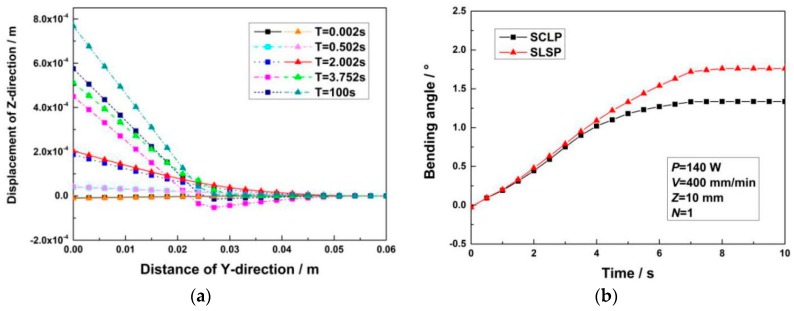
Comparison of Z-direction displacements between the SCLP and the SLSP: (**a**) Comparison of Z-direction displacements along Y-direction at different times; (**b**) comparison of bending angles at the midpoint of the free end at different times.

Figure 11a shows the Z-direction displacement distributions of the SCLP (square mark) and the SLSP (triangular mark) in the Y-direction at different times. Under the action of the laser beam, the SCLP and the SLSP produce negative bending due to thermal expansion. Because the small plastic zone of the SCLP results in a small amount of expansion, the negative bending angle of the SCLP is smaller than that of the SLSP. As the laser beam scans, the SCLP changes from negative bending to positive bending, causing the Z-direction displacement of the free end to increase. During laser bending, not only the carbon steel layer of the SCLP accelerates the heat conduction, but also its mechanical properties make it difficult to deform. As a result, the positive bending angle of the SCLP is also smaller than that of the SLSP. Figure 11b shows the curves of bending angles of the SCLP and the SLSP changing with time. It can be observed that the bending angles of the SCLP and the SLSP produced by each pulse are obviously increased. During cooling, due to residual heat, the bending angle slowly increases to reach a certain extent before staying constant. The final bending angles of the SCLP and SLSP are 1.336° and 1.760°, respectively.

## 4. Results and Discussions

**Figure 12:**
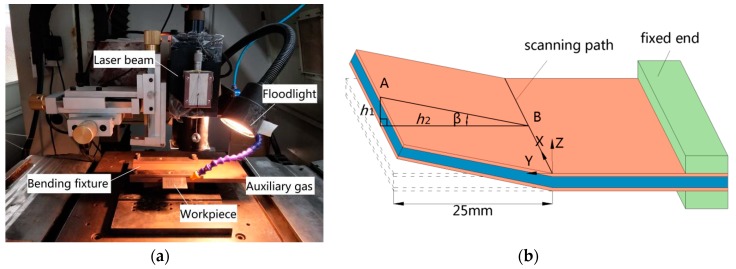
Experimental devices of laser bending: (**a**) Machine of laser bending; (**b**) schematic diagram of laser bending.

In order to verify the accuracy of the simulation, the laser bending experiment is operated on an LMT-5040 precise numerical control machine using a JK701H Nd:YAG pulsed laser device for the SCLP and SLSP with different process parameters as shown in Figure 12a. As shown in Figure 12b, the workpiece (with the size of 60 mm × 50 mm × 1 mm) is single-end clamped with the free end 25 mm away from the scanning path. There are two criteria for the selection of laser parameters. One is obvious bending of the SCLP, and the other is no ablation phenomenon on the surface of the SCLP. Meanwhile, considering the interaction of other energy parameters, the ranges of process parameters are shown in Table 1. In order to measure the bending angle of the plate, the tangent calculation method is used through the measurement of three coordinates measuring system (with the precision of 1 μm) as below:

(19) $$\beta \;=\;\arctan\left(h_{1}/h_{2}\right)$$

where *β* is the bending angle of the SCLP, *h*_1_ is the projection of AB in the *Z*-axis direction, and *h*_2_ is the projection of AB in the *Y*-axis direction.

Compared with the experimental results, the simulated errors are expressed as follows:

(20) $$\delta_{1}\;=\;\left(T_{1}-T_{2}\right)/T_{2}$$

(21) $$\delta_{2}\;=\;\left(\beta_{1}-\beta_{2}\right)/\beta_{2}$$

where *T*_1_ and *T*_2_ are the temperatures of simulation and experiment, respectively; *β*_1_ and *β*_2_ are the bending angles of simulation and experiment, respectively; and *δ*_1_ and *δ*_2_ are the errors of temperature and angle between simulation and experiment, respectively.

**Figure 13:**
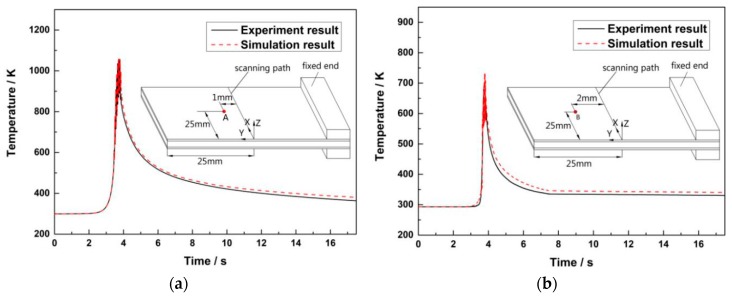
Temperature variations of measuring point: (**a**) Measuring point 1; (**b**) measuring point 2.

In terms of temperature field, the simulation results are compared with the experimental verifications as shown in Figure 13 for the SCLP. The process parameters are as follows: *P* = 140 W, *V* = 400 mm/min, *Z* = 10 mm, *N* = 1. In Figure 13a,b, the measuring point is placed 1 mm and 2 mm from the scanning line, respectively. The simulation results and experimental verifications follow the same trend, and the maximum of the temperature errors is 5.63%.

**Figure 14:**
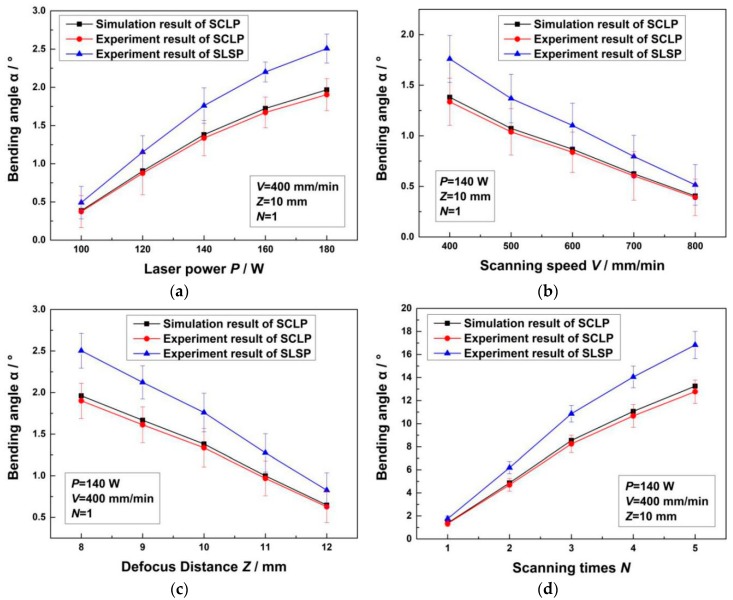
Comparison of bending angle curves of the SCLP and the SLSP with different process parameters: (**a**) Laser power; (**b**) scanning speed; (**c**) defocus distance; (**d**) scanning time.

In terms of the displacement field, Figure 14 shows the bending angles of the SCLP and the SLSP with different laser powers, scanning speeds, defocus distances and scanning times. Specifically, Figure 14a shows that with the increase of laser power, the temperature gradient of the SCLP increases, which causes the extension of the plastic zone with the temperature exceeding the recrystallization temperature. On account of the steepening vertical strain gradient in the thickness direction, the bending angle of the SCLP becomes larger. Figure 14b demonstrates that the increase in scanning speed produces two results: reductions of the overlap rate of the laser spot and the duration of laser heating. The decrease of energy density decreases the temperature gradient in the thickness direction and thermal stress, leading to a decrease of the bending angle. Figure 14c indicates that with the increase of defocus distance, the energy density at the center of laser spot decreases, which causes the decreases of the temperature gradient and the depth *H* of the plastic zone so that the bending angle decreases. In Figure 14d, the superposition of scanning time extends the process time. Hence, the bending angle generated by every laser scan is visibly increased after superposition. It also can be seen from Figure 14 that the bending angle of the SCLP is smaller than that of the SLSP with the same process parameters, which is consistent with the analyses of temperature field, stress field, strain field and displacement field.

**Figure 15:**
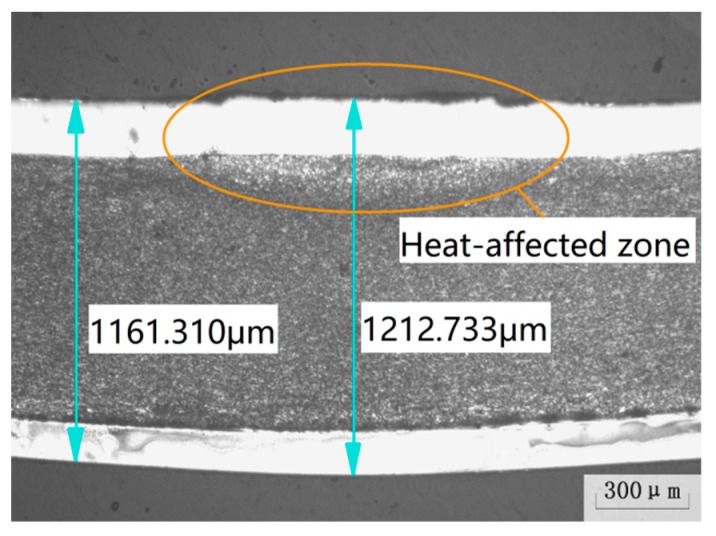
Thickening phenomenon of the SCLP.

Moreover, it is discovered that experimental verifications of the bending angle are slightly smaller than the simulation results in Figure 14. Merklein et al. [14] summarized the possible reason that dislocation structures in the plastic zone change mechanical properties of the material, resulting in the reduction of bending angle during the laser bending process. Furthermore, Figure 15 shows that there is a material thickening phenomenon in the heat-affected zone. Due to material accumulation, the direction of stress is opposite to the bending direction of the SCLP. Due to this counteraction, the model value is larger than the actual bending angle. Under the condition of different parameters, the maximum error of Chi’s simulation results [15] is 4.93%, while the maximum bending angle error is 3.74% in this paper. The accuracy of the simulation is improved by 1.19%. Therefore, numerical simulation of SCLP considering the thickness of the interface accurately predicts bending angles during the laser bending process, which is of great significance for analyzing the bending mechanism of the SCLP.

## 5. Conclusions

Based on ANSYS and electron probe analysis, the deformation behavior of the SCLP during pulsed laser bending is investigated using a multi-layer FEM including 7 μm thick transition layers. Through the analysis on temperature field, stress field, strain field and displacement field of the SCLP along the thickness direction, the impacts of transition layer and carbon steel layer on bending angle are explored. The conclusions are as follows:

1. During the laser bending process, due to the interfacial thermal resistance of the transition layer and rapid thermal conduction of the carbon steel layer, the temperature of the upper stainless steel layer decreases rapidly in the thickness direction, and the value is from 1534.81 K to 852.997 K. As a result, based on the temperature gradient mechanism considering the depth of the plastic zone, the depth of the plastic zone of the SCLP is smaller than that of the SLSP.
2. The compressive stress and strain of the SCLP in the plastic zone are smaller than those of the SLSP, resulting in a smaller bending angle of SCLP. When the laser power is 140 W, the scanning speed is 400 mm/min, the defocus distance is 10 mm, and the scanning time is 1, the bending angle of the SCLP is 1.336°, which is smaller than the bending angle 1.760° of the SLSP.
3. The bending angle of the SCLP increases with an increase in laser power *P* and scanning time *N*, and decreases with the increase in scanning speed *V* and defocus distance *Z*. With the same process parameters, the maximum error of bending angles is 3.74%. The simulation accurately predicts bending deformation of the SCLP during the process of laser bending.

## Figures and Tables

**Table 1:** Simulation conditions under different laser parameters.

Classification	Process Parameters	Unit	Value Ranges
Process parameters	Power *P*	W	100, 120, 140, 160, 180
	Scanning speed *v*	mm·min^−1^	400, 500, 600, 700, 800
	Defocus distance *Z*	Mm	+8, +9, +10, +11, +12
	Scanning number *N*		1, 2, 3, 4, 5
Laser parameters	Laser frequency *f*	Hz	40
	Pulsed width *t*_p_	Ms	2
	Wavelength λ	μm	1.064
